# Biochemical Characterization of AGMO Variants Implicated in Relapses in Visceral Leishmaniasis

**DOI:** 10.1093/infdis/jiy090

**Published:** 2018-02-23

**Authors:** Katrin Watschinger, Markus A Keller, Georg Golderer, Stefan Coassin, Johannes Zschocke, Ernst R Werner

**Affiliations:** 1 Division of Biological Chemistry, Biocenter, Medical University of Innsbruck, Austria; 2 Division of Human Genetics, Medical University of Innsbruck, Austria; 3 Division of Genetic Epidemiology, Department of Medical Genetics, Molecular and Clinical Pharmacology, Medical University of Innsbruck, Austria

**Keywords:** Alkylglycerol monooxygenase, genetic variants, relapses in leishmaniasis

To the Editor—Marquet et al [1] recently described 2 variants in *AGMO* associated with relapses in visceral leishmaniasis (*kala-azar* [KA]) in Sudanese children. This gene codes for the ether lipid–cleaving enzyme alkylglycerol monooxygenase (AGMO), a membrane-bound enzyme expressed in a variety of tissues and cell lines [2] but also in primary murine macrophages, where it is differentially regulated in inflammatory and antiinflammatory priming of the cells [3]. Despite conflicting reports about the impact of AGMO on lyso-PAF and PAF levels by Tokuoka et al [4] and us [3], both groups provided consistent evidence for the regulation of AGMO in primary macrophages, implying a yet to be revealed role for this enzyme in immunity.

Such a role was described by Marquet et al [1], through their identification of 2 *AGMO* variants: (1) a missense variant in exon 7 (c.701A>G, rs143439626, p.Lys234Arg, K234R) and (2) a nonsense variant in exon 12 (c.1213C>T, rs139309795, p.Arg405*, R405X). In all families in this study, the 2 alleles cosegregate (data for individual 5 in family 1 are most likely a genotyping error) and, thus, are positioned on the same allele in a *cis* configuration. This is in line with the highly similar allele frequency of the 2 variants in relevant populations, with the exception of individuals of East Asian descent, where p.Lys234Arg appears to occur without p.Arg405* (available at: http://gnomad.broadinstitute.org). Indeed, these 2 rare *AGMO* variants are reported to be in linkage disequilibrium (LD) in an individual from the Kenyan LWK population in the 1000 Genomes data set [5] (pairwise LD r^2^ = 1, Dʹ = 1; available at: http://www.ensembl.org). Therefore, the genotypes in the pedigrees consist of 1 or 2 copies of the p.[Lys234Arg;Arg405*] allele (c.[701A>G;1213C>T] at the nucleotide level). This allele appears to have a frequency of 0.18 in the African population, indicating that about 1 in 275 Africans are heterozygous for this allele.

Marquet et al extracted both *AGMO* variants from whole-exome-sequencing data by applying different filters, including a UMD-predictor to predict potential pathogenicity. We doubted the predicted deleteriousness of the p.Lys234Arg variant, since bovine AGMO carries an arginine in this position (NP_001179902.1).

We performed a study by using methods that accorded with those reported by Watschinger et al [6], with the following adaptations. The template for site-directed mutagenesis was untagged human AGMO (NM_001004320.1). HEK293T were transfected using Turbofect (Thermo Scientific), following the manufacturer’s protocol. For membrane preparation, HEK293T cells were harvested as dry pellet. For the AGMO activity assay, the cells were harvested and shock frozen in 0.5% CHAPS and 1 mM dithioerythritol.

For Western blots, AGMO was stained with TMEM195 rabbit polyclonal antibody (dilution, 1:1000; Proteintech) and goat anti-rabbit CyTM5 (1:2500; GE Healthcare). Actin was used as visual loading control and stained with mouse anti-actin antibody (dilution, 1:1500; Millipore) and goat anti-mouse CyTM3 (1:1250; GE Healthcare). Blots were scanned using a red laser (633 nm excitation and 670 nm emission; BP30) for Cy5 and a green laser (532 nm excitation and 580 nm emission; BP30) for Cy3. Blot signals were quantified by ImageQuant TL software (GE Healthcare) and divided by the respective amount of protein applied to the gel (12.5–30 µg). Statistical analysis was performed with GraphPad Prism 7.03, using 1-way analysis of variance, followed by the Tukey multiple comparisons test.

To experimentally test the effects of the variants, we cloned the 2 variants alone and in combination into an AGMO expression plasmid and analyzed protein expression and cellular AGMO activity. As shown in Figure 1, variant p.Lys234Arg (KR) did not change both parameters. Variant p.Arg405* (RX), in contrast, significantly reduced both protein expression (to 40.8% of the wild type value; *P* = .0004) and cellular activity (to 17.7%; *P* = .001). Thus, the truncated variant showed reduced catalytic activity per synthesized protein, in addition to diminished protein expression. For the double-mutant p.[Lys234Arg;Arg405*], protein expression decreased to a level similar to that for p.Arg405* alone (to 39.3% of the wild type value), but cellular activity tended to be further suppressed (to 5.33%), although this was not significantly different from observations for p.Arg405* alone (*P* = .78). Based on the data from Marquet et al [1], cosegregation of the p.Lys234Arg variant with the p.Arg405* variant explains why p.Lys234Arg, which, on its own, is functionally silent, was interpreted to be associated with the disease.

**Figure 1.**
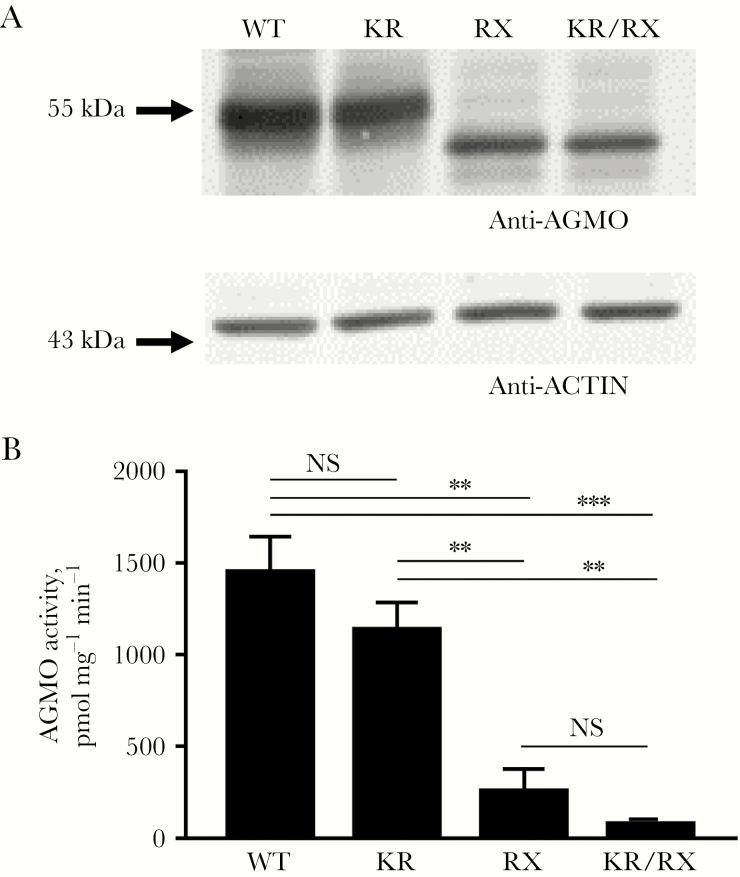
Analysis of cellular alkylglycerol monooxygenase (AGMO) activity and protein expression of wild-type (WT) AGMO versus mutant p.Lys234Arg (KR), mutant p.Arg405* (RX), and double mutant p.[Lys234Arg;Arg405*] (KR/RX) AGMO. *A*, Protein expression of WT, KR, RX, and KR/RX AGMO was analyzed by Western blot in microsomal fractions after transfection in HEK293T cells. The upper panel shows the AGMO signal, and the lower panel shows the signal of actin, the loading control. Marker protein sizes are indicated as arrows. Representative Western blots are shown. *B*, Cellular AGMO activity was measured in parallel samples, using our sensitive fluorescent high-performance liquid chromatography AGMO microassay [7]. Results are means ± standard errors of the mean for 3 independent experiments. NS, not significant. **P < .01 and ***P < .001.

Of 9 individuals who contracted KA in the study, 2 who experienced relapse (and were from the same family) were homozygous for the variant allele, and 3 were heterozygous; the genotype in 1 needs to be reassessed. Three individuals who did not show relapse were homozygous for the wild-type allele; the relevance of the controls without KA is doubtful. While the observations are compatible with a role of AGMO deficiency in KA relapse, the study does not provide adequate statistical support to confirm this association; no probability has been calculated that reasonably excludes the possibility that this finding was due to chance alone. Further work is required to confirm a possible association between AGMO loss of function alleles and KA relapse. In any event, our data provide experimental evidence for the assumed deleteriousness of variant p.Arg405* co-segregating with the enzymatically silent variant p.Lys234Arg.

## Notes

***Acknowledgments.*** We thank Petra Loitzl, Rita Holzknecht, and Nina Madl, for expert technical assistance.

***Financial support.*** This work was supported by the Austrian Science Fund (FWF): projects P28769 [to E. R. W.] and P30800 [to K. W.].

***Potential conflicts of interest.*** All author: No reported conflicts of interest. All authors have submitted the ICMJE Form for Disclosure of Potential Conflicts of Interest. Conflicts that the editors consider relevant to the content of the manuscript have been disclosed.
